# Triple Therapy in COPD: Can We Welcome the Reduction in Cardiovascular Risk and Mortality?

**DOI:** 10.3389/fmed.2022.816843

**Published:** 2022-03-23

**Authors:** Paolo Solidoro, Carlo Albera, Fulvia Ribolla, Michela Bellocchia, Luisa Brussino, Filippo Patrucco

**Affiliations:** ^1^Division of University Respiratory Medicine, Cardiovascular and Thoracic Department, AOU Città della Salute e della Scienza di Torino, Turin, Italy; ^2^Department of Medical Sciences, University of Turin, Turin, Italy; ^3^Allergy and Clinical Immunology Unit, AO Ordine Mauriziano Hospital, Turin, Italy; ^4^Division of Respiratory Diseases, Medical and Specialistic Department, AOU Maggiore della Carità, Novara, Italy; ^5^Translational Medicine Department, University of Piemonte Orientale, Novara, Italy

**Keywords:** chronic obstructive pulmonary disease (COPD), triple inhaled therapy, ICS, LAMA, LABA, CV mortality

## Abstract

Chronic obstructive pulmonary disease (COPD) is a complex disease which consists in the reduction of the airflow and leads to the disruption of the pulmonary tissue due to a chronic inflammation. The progression of the disease is characterized by an exacerbation of the symptoms and the presence of life-threatening systemic complications, such as stroke and ischemic heart disease, with a progressive decline in lung function which can deeply impact the quality of life. Mortality represents the most important COPD outcome, with an increased risk in patients with cardiovascular comorbidities. The efficacy and safety of triple inhaled therapy were demonstrated by numerous controlled trials. Above all, many robust data are now available on the effectiveness of the triple therapy to reduce mortality in COPD patients.

## Introduction

Chronic obstructive pulmonary disease (COPD) is a slowly progressive (1) complex disease, which consists in the reduction of the airflow and leads to the disruption of the pulmonary tissue due to a chronic inflammation (2, 3). According to the Global Initiative for Chronic Obstructive Lung Disease (GOLD) initiative, the airflow limitation “is usually both progressive and associated with an abnormal inflammatory response of the lungs to noxious particles or gases” (4). Over the lifetime, COPD might predispose to exacerbations and serious illness (5). According to the World Health Organization (WHO), 251 million cases of COPD were globally reported in 2016 and it is estimated that 3.17 million deaths were caused by the disease in 2015 (5). Moreover, COPD prevalence is likely to increase in the coming years due to higher smoking habits and aging populations (5).

Although the development of COPD might be considered multifactorial (6), smoking can be considered the most important environmental risk factor in the development of COPD (7), leading to a rapid decline in lung function and an increase in mortality in smokers compared to non-smokers (8). However, multiple risk factors might be involved in the development of the disease which can be classified into environment-related risk factors and patient-related risk factors, such as respiratory infections, occupational exposures, ambient air pollution, passive smoke exposure, and diet. Moreover, some other genetic factors can be involved in the establishment of COPD, such as the Alpha1-antitrypsin deficiency (9).

Another important risk factor is reduced Daily Physical Activity (DPA), which might be considered an important cause of cardiovascular (CV) morbidity (10).

COPD pathophysiology is also influenced by toxic particles inhalation (11), which indeed may lead to chronic inflammation that might exacerbate the COPD prognosis (12). These substances act by activating both epithelial cells and alveolar macrophages, which are involved in the release of proinflammatory cytokines and proteases, such as matrix metalloproteinases (MMPs) and neutrophil elastase. The release of neutrophil elastase leads to elastin degradation and the alveolar wall destruction (11).

COPD clinical course is characterized by persistent and productive cough, dyspnea, and chest tightness (1). The progression of the disease is characterized by an exacerbation of the symptoms and the presence of life-threatening systemic complications (1), such as stroke and ischemic heart disease (13), which are associated with a progressive decline in lung function, and can deeply impact the quality of life (14).

Acute Exacerbations of COPD are mainly triggered by respiratory infections, environmental factors (15), or both (14). Moreover, disorders of the immune system may increase the frequency of AECOPDs, which are associated with an increase of airway inflammation, mucus hypersecretion and gas trapping, and are characterized by both minor (wheeze, sore throat, cough, fever, chest tightness, fatigue, sleep disturbance) and major symptoms (dyspnea, increased sputum volume and purulence) (14). Often, after recurrent exacerbations, respiratory failure might occur, due to hypoxia which is usually characterized by the dropping of the blood oxygen levels during rest, sleep, or activity (16).

## Mortality and Cardiovascular Risk in COPD

Mortality represents the most important COPD outcome (17), even though this event is often underestimated (18), or underreported (19). With 3.23 million deaths in 2019, COPD represents the third cause of death worldwide (20). In the last years, mortality for COPD rapidly increased, particularly among old women, with an increase of three-fold higher among women ≥75 years old (21). This could be due to the high susceptibility to secondary COPD complications, but also to the high smoking prevalence among the global population (22). To date, in Italy 3.5 million people suffer for COPD, with approximately 55% of death due to the respiratory disease. However, the prevalence of the disease is high and often variable between Italian regions, and is related to a late diagnosis after hospitalization for exacerbations, while mild-to-moderate disease are often underdiagnosed. The national PNE (Programma Nazionale Esiti) estimated a high number of hospitalizations for COPD (>100.000/year), with a rate of mortality of 9.8% and 13.45% hospital readmissions at 30 days (https://pne.agenas.it/).

Multiple risk factors may contribute to increase the mortality risk in patients with COPD.

In particular, the reduction in the forced expiratory volume in one second (FEV_1_) is a strong risk factor of all-cause mortality in moderate COPD (23, 24).

Fletcher and Peto for the first time demonstrated that the initial value of measured FEV_1_ correlated with the increased risk of death in patients with COPD (25).

Since COPD patients are frequently affected by other comorbidities, cardiovascular disease and malignancy are still the predominant causes of death. Moreover, AECOPDs represent another important risk factor which may influence the mortality rate (26).

Nowadays, multidimensional indexes including BODE, ADO (age, dyspnea, airflow obstruction) and DOSE (dyspnea, obstruction, smoking, exacerbation) indexes, have been used in order to predict the risk of mortality in COPD patients. Several papers, underline this increased risk as also associated with low exercise capacity and increased number of exacerbations (27, 28).

The decrease of morbidity and mortality in COPD is one of the main objectives of GOLD initiative, which represents the most important guide for the management and staging of COPD (29). Last updates, matched spirometry staging, symptoms and exacerbations in the ABCD Classifications in order to choose the best treatment approach (30).

Indeed, from 2015 to 2019, mortality was taken into account was reconsidered because of similar variability between ABCD groups (30).

Cardiovascular diseases (CVD) represent the most frequent cause of death worldwide (29) and it is an important comorbidity in the prediction of all-cause mortality in COPD patients (31). This could be due to the risk shared among two diseases, such as old age, smoking history and increased systemic inflammation (32, 33).

Ischemic heart disease, heart failure and cardiac arrhythmias are the most commonly observed CVDs in COPD patients (34). Numerous mechanisms might be involved in the development of COPD-related CVD, including lung hyperinflation, hypoxemia, pulmonary hypertension (PH), systemic inflammation, oxidative stress, and exacerbations.

Hyperinflation is the major cause of COPD mortality (35) and it deeply impacts the respiratory system. As a matter of the fact, hyperinflation results from the destruction of the lung parenchyma and subsequent loss of lung elasticity, or it occurs when the air becomes trapped during the subsequent respiratory cycle (36), being the trigger of dyspnea (37). The direct consequence is an airflow limitation which causes increased pressure in the cardiopulmonary system, right-ventricular dysfunction, impaired left-ventricular filling and reduced cardiac output (38). Moreover, the airflow limitation results in a mismatch between ventilation and perfusion, which contributes to the development of hypoxemia (39). Finally, the increase of intrapulmonary pressure can lead to the dilation and hypertrophy of the right ventricle (RV), causing the septum displacement to the left ventricle, with compromission of ventricular filling, stroke volume (SV) and cardiac output (40, 41). Moreover, hypoxemia is responsible for pulmonary vasoconstriction, which results in a diastolic disfunction in the right ventricle (42).

Ventricular arrhythmias are common in COPD patients, especially during exacerbations (43) and include supraventricular or ventricular premature beats, atrial fibrillation, atrial flutter, multifocal atrial tachycardia, supraventricular tachycardia, and non-sustained ventricular tachycardia (44).

**Figure 1:**
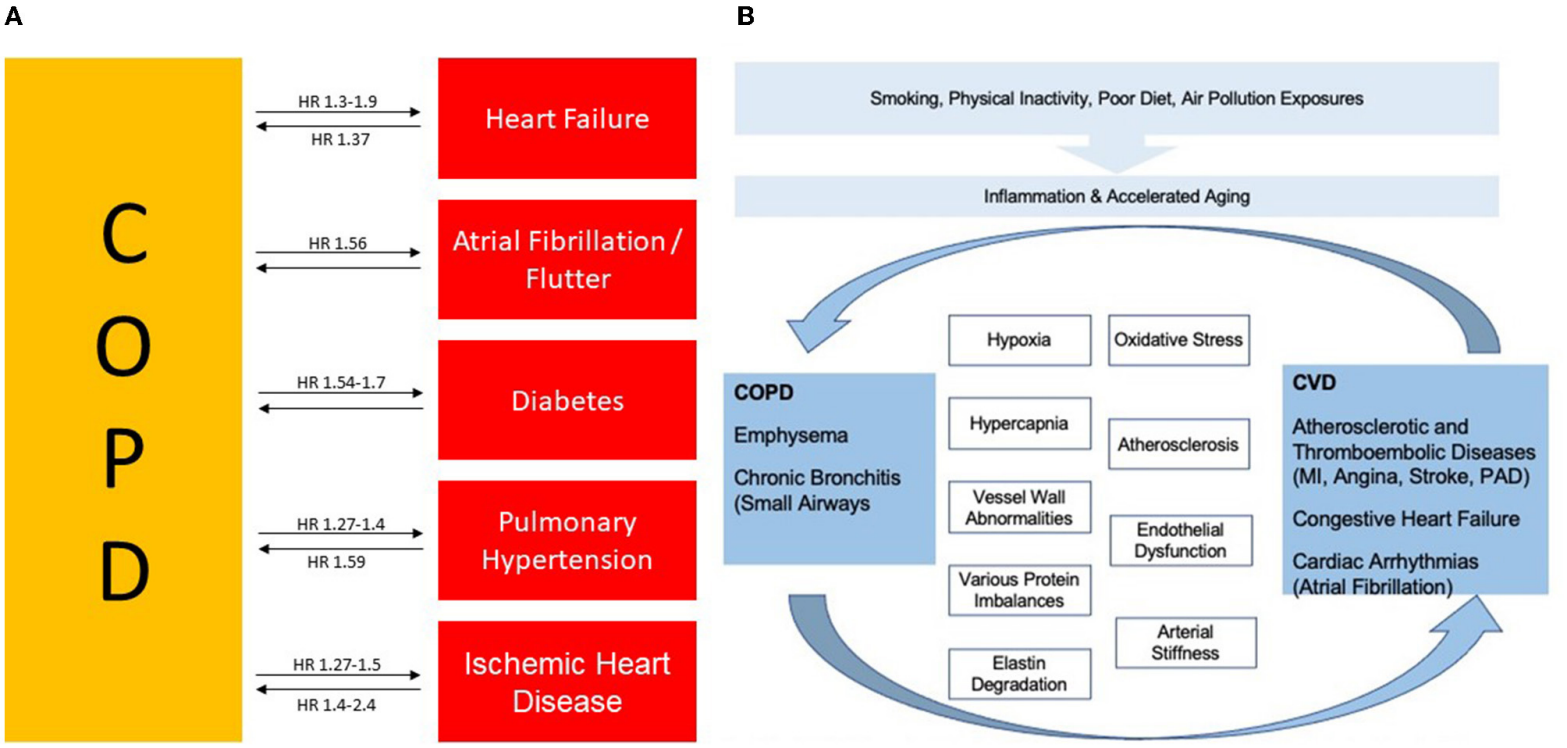
**(A)** Association between COPD, cardiovascular disease and risk factors and their interrelationship. **(B)** Both diseases are closely linked for several features. COPD, Chronic Obstructive Pulmonary Disease; CVD, Cardiovascular Disease, HR, Hazard Ratio; MI, Myocardial Infraction; and PAD, Peripheral Arterial Disease. Figure adapted from Rabe et al., (38) and adapted from Morgan et al., (34).

The mechanism responsible for the increased risk of cardiovascular disease in patients with COPD is not known; however, several processes have been proposed as alternatives, which are involved in the pathogenesis of cardiovascular disease, playing an important role in driving the increased cardiovascular risk associated with enhanced mortality in COPD (45). Indeed, several biomarkers demonstrated a prognostic value for COPD such as C-reactive protein, fibrinogen, brain type natriuretic peptide (BNB), N- terminal pro-BNP (NT Pro BNP), troponin, VEGF, surfactant protein D and the neutrophil/lymphocyte ratio (NLR) (46, 47). Figure 1 summarizes the link and the possible mechanism between COPD and CV risk.

The role of medications for cardiovascular diseases needs to be fully understood. A concomitant COPD treatment with angiotensin-converting enzyme inhibitors (ACEIs), angiotensin receptor blockers (ARBs), β-blockers and statins have been correlated with a better management of the disease (48). On the other hand, some COPD treatments might increase CV risk (49–52), such as methylxanthines (53) (which have been now withdrawn), β-agonist drugs (54), inhaled short-acting β-agonists (31), inhaled long-acting β-agonists and anticholinergic drugs (short and long acting) (54). Single dose of indacaterol (55) reduces lung hyperinflation in acute conditions, with a clinically relevant improvement of dyspnea in patients with COPD and CV pathology.

These modifications are associated with a significant increase of the right ventricular compliance indexes and may have a role in improving left ventricular preload leading to a reduction in cardiac frequency (55). In 2018, Hohfòeld et al. (56) conducted a s double-blind, randomized, two-period crossover, placebo-controlled, single-center study aimed to evaluate whether dual bronchodilation with indacaterol/glycopirronium improved cardiac function. This dual bronchodilation significantly improved cardiac function as measured by left-ventricular end-diastolic volume (56).

Systemic corticosteroids are often administered in COPD patients during exacerbations. Schols et al. (57) conducted a study aimed to evaluate the role of systemic corticosteroids, administered orally, in the CV risk for COPD patients. In this study, an oral glucocorticoid use (10 mg/day) was associated with an increased risk, which was even higher when the administered dose has been increased. In conclusion, maintenance treatment with oral glucocorticoids was associated with increased mortality in a dose-dependent manner (57).

## Triple Inhaled Therapy

Nowadays, COPD treatment follows a stepwise approach, where a different treatment regimen is indicated for each class (15). Patients belonging to group A present one or fewer exacerbations, without hospitalizations, and not severe symptoms. In this case, patients should receive a short- or long-acting bronchodilator. Group B patients present one or fewer exacerbations, without hospitalization, but are characterized by severe symptoms. A long-acting bronchodilator, either a long-acting muscarinic antagonist (LAMA) or long-acting β 2-agonist (LABA) are indicated for these patients. Group C includes patients with two or more exacerbations which can lead to hospitalization; they do not present severe symptoms and they should be treated with LAMA as first-line therapy. Finally, group D includes symptomatic patients with an history of two or more exacerbations with the need of hospitalization. For these patients, the first-line therapy is a LAMA and for patients with very severe symptoms (COPD Assessment Test Score [CAT] greater than or equal to 20) a LAMA/LABA combination is recommended (58). Moreover, when the eosinophil count is above the threshold of ≥300 eosinophils/μl, a combination on LABA/ICS is strongly recommended as first approach (15).

GOLD recommends triple inhaled therapy for patients who experience recurrent exacerbations despite treatment with either a dual bronchodilator or LABA/ICS combination (59). Triple inhaled therapy includes an inhaled corticosteroid (ICS), a long-acting β 2-agonist (LABA) and a long-acting muscarinic antagonist (LAMA) (58). Indeed, it can improve FEV1 and health status (60).

Nowadays, there are three available inhaled ICS/LABA/LAMA fixed-dose combinations (FDCs): Budesonide/Formoterol/Glycopyrronium (61), Beclometasone/Formoterol/Glycopyrronium (62) and Fluticasone Furoate/Vilanterol/Umeclidinium (63). The three available triple combinations are delivered by three different devices: the aerosphere MDI (61), the Ellipta DPI (63) and a MDI (64). Inhaled ICS/LABA/LAMA FDCs are characterized by different mechanisms of action which provide benefits on exacerbations' reduction, lung function and health status improvement as well by reducing the risk of death (compared with increasing the dose of the single bronchodilator) (64).

Corticosteroids bind the C-terminal of glucocorticoid receptor which is localized in the cytoplasm of target cells. Corticosteroids directly regulate 10–100 genes per cell, but the interaction with other transcription factors indirectly regulates many other genes. The most anti-inflammatory effect of ICS is the inhibition of inflammatory proteins' synthesis, such as cytokines 18. Moreover, ICS increase their effectiveness by inducing the expression of β2-adrenoreceptors, and human genes for β2 receptors have three potential glucocorticoid response elements (65). For this reason ICS can positively impact the cardiovascular outcomes through the reduction of serum CRP levels (66).

LAMAs act by blocking the endogenous cholinergic tone. In particular, vascular innervation of the airways results in the contraction of the smooth bronchial musculature. Acetylcholine can play different roles in this function, being responsible both as a neurotransmitter at the ganglia level, a function mediated through M1 muscarine receptors, and as a neurotransmitter in the neuromuscular junction, resulting in the contraction of smooth musculature through the muscarinic receptor M3 (67). The M2 receptors, located in the post-gangliary neuron, inhibits the release of acetylcholine. Anticholinergic bronchodilators have been designed to prevent contraction of smooth muscles primarily by blocking the M3 receptor in the neuromuscular junction (67).

Beta2-agonist induce smooth bronchial muscles relaxation through their action on beta2-adrenergic receptors. These receptors, located in the bronchial smooth musculature surface, consist of G-proteins which are able to increase the adenosine monophosphate levels, resulting in the activation of the protein kinase A and in the subsequent relaxation of the smooth bronchial musculature (68).

Combining each family molecule in a single inhaler is the easiest way to deliver triple therapy (69). This therapeutic approach demonstrated a significant reduction in AECOPDs and hospitalizations, as well as a reduction of all-cause mortality (70).

The efficacy and safety of inhaled ICS/LABA/LAMA FDCs in patients with COPD have been evaluated in several clinical trials.

TRIBUTE was a randomized, parallel-group, double-blind study that aimed to compare a single-inhaler triple combination of beclometasone dipropionate, formoterol fumarate, and glycopyrronium (BDP/FF/G) vs. a single-inhaler dual bronchodilator combination of indacaterol plus glycopyrronium (IND/GLY), evaluating the rate of moderate-to-severe AECOPDs over 52 weeks of treatment. Eligible patients were 40 years or older, current or ex-smokers, with a diagnosis of COPD, a FEV_1_/FVC post-bronchodilator (salbutamol 400 μg) ratio of less than 0·7, and severe or very severe airflow limitation (FEV_1_ <50%). Patients had at least one moderate or severe COPD exacerbation in the previous 12 months, a COPD Assessment Test total score of at least 10 and, for at least 2 months before screening had used an inhaled corticosteroid plus a long-acting β2-agonist, an inhaled corticosteroid plus a long-acting muscarinic antagonist, a long-acting β2-agonist plus a long-acting muscarinic antagonist, or long-acting muscarinic antagonist monotherapy, but not triple therapy. 1532 patients were enrolled among 17 countries and randomized 1:1 to receive 52 weeks of treatment with two inhalations of extrafine BDP/FF/G (87 μg/5 μg/9 μg) twice per day (764 patients) or one inhalation of IND/GLY (85 μg/43 μg) per day (768 patients). The primary endpoint was the rate of moderate-to-severe COPD exacerbations across 52 weeks of treatment. Results showed a rate of moderate-to-severe exacerbation of 0.50 per patient per year (95% CI 0.45–0.57) for BDP/FF/G group and 0.59 per patient per year (0.53–0.67) for IND/GLY group. Adverse events were reported by 490 of 764 patients (64%) receiving BDP/FF/G and 516 of 768 (67%) patients receiving IND/GLY (71). Additional analyses on mortality were assessed by Vestbo et al. in a pooled analysis for all extrafine ICS-containing treatments. The rate of fatal events was 2.1% in the BDP/FF/G and 2.7% in the IND/GLY group. However, no statistical significance in the reduction in the risk of death was observed within 1 year of observation (72).

The IMPACT trial was a randomized study involving 10,355 patients with symptomatic COPD (CAT>10; FeV_1_ <80%) and a history of moderate/severe exacerbations. Patients were randomized to receive fluticasone furoate/umeclidium/vilanterol (FF/UMEC/VI) (100 μg/62.5 ug/25 ug) OR fluticasone furoate/vilanterol (FF/VI) (100 ug/25 ug) OR umeclidinium/vilanterol (UMEC/VI) (62.5 ug/25 ug). Results showed that the reduction of moderate to severe COPD exacerbations rate was significantly greater for triple therapy with FF/UMEC/VI group than fluticasone furoate-vilanterol or umeclidinium-vilanterol groups, with rates of 0.91 per year, 1.07 per year and 1.21 per year, respectively. The COPD-related hospitalization rate was lower in the triple therapy group than umeclidinium-vilanterol (0.13 vs. 0.19). Moreover, a significant reduction in all-cause mortality (ACM) risk was demonstrated in patients treated with FF/UMEC/VI vs. UMEC/VI, with a hazard ratio of 0.58 (73). In 2020, a *post-hoc* analysis was performed to report any ACM and the impact of stepping down therapy using the additional vital status data. Results showed a lower rate of death in patients treated with FF/UMEC/VI (2.36%) compared to both FF/VI (2.64%) and UMEC/VI (3.19%) (74). Time to ACM was also evaluated, including off-treatment data: the hazard ratio for patients treated with FF/UMEC/VI was 0.72 compared with UMEC/VI group, and was 0.82 for patients treated with FF/VI compared to UMEC/VI ones. Because of the randomization, 40% of the patients were stepped down to FF/VI and 20% were stepped down to UMEC/VI. Results demonstrated a reduced risk of on/off-treatment death for patients who were maintained on a triple therapy compared to those who underwent step down to one of the dual therapies (FF/VI or UMEC/VI) (hazard ratio of 0.71 compared with patients stepped down to FF/VI; hazard ratio of 0.62 compared to patients who stepped down to UMEC/VI). It is worth to note that 76.9% of the patients were treated with a therapeutic regimen including an ICS at the time of enrollment: in these patients, mortality was lower if they were maintained on an ICS-containing regimen compared with UMEC/VI (74).

The ETHOS (75)study was a phase 3 randomized trial that evaluated efficacy and safety of triple therapy at two dose levels of inhaled glucocorticoid over 52 weeks. The study included 40–80 years old patients with symptomatic COPD (defined as a score of ≥10 on the COPD Assessment Test, on which scores range from 0 to 40, with higher scores indicating more symptoms; the minimum clinically important difference is 2 points). Patients were receiving at least two inhaled maintenance therapies at the time of screening, with a post-bronchodilator FEV_1_/FVC ratio of <0.7, and a postbronchodilator FEV_1_ of 25 to 65% of the predicted normal value. Patients were smokers or ex smokers and had a documented history of at least one moderate or severe COPD exacerbation (if their FEV_1_ was <50% of the predicted normal value) or at least two moderate or at least one severe COPD exacerbation (if their FEV_1_ was ≥50% of the predicted normal value) in the year before screening. 8,509 patients were included in the study and randomly assigned to the groups receiving: inhaled budesonide/glycopyrronium/formoterol, 320 μg/18 ug/ 9.6 ug (320-BGF; 2,137 patients); inhaled budesonide/glycopyrronium/formoterol, 160 ug/18 ug/ 9.6 ug (160-BGF; 2,121 patients); glycopyrronium/formoterol-18 ug/9.6 ug- (GFF; 2,120 patients); budesonide/formoterol-320 ug/9.6 ug (BFF; 2,131 patients). The primary endpoint was to assess the annual rate of moderate or severe COPD exacerbations. BGF triple therapy resulted in a lower rate of moderate or severe COPD exacerbations (1.08 and 1.07 for the 320 ug and 160 ug, respectively) than GFF (1.42) or BFF (1.24) (75). Moreover, the rate was significantly lower with 320- μg–budesonide triple therapy group than with GFF (24% lower) or BFF ones (13% lower). Mortality was evaluated as key secondary endpoint, and a lower risk of death from any cause was observed only in the 320-μg–budesonide triple-therapy group (75). Overall, the risk of death from any cause was 46% lower for 320-μg–budesonide triple therapy vs. glycopyrronium- formoterol (75). Since 384 of 8,509 patients were missing vital status at Week 52 in the original analyses, a *post-hoc* analysis was performed to evaluate the robustness of the ETHOS mortality findings. Results showed that the risk of death with BGF 320 was significantly lower than GFF (with a hazard ratio of 0.51), and there were no significant differences in mortality when comparing BGF 320 to BFF, although numerical reductions of 28% were observed. Deaths from cardiovascular causes occurred in 0.5%, 0.8%, 1.4%, and 0.5% of patients in the BGF 320, BGF 160, GFF, and BFF groups, respectively (76). Overall, the risk of death was significantly lower (49%) with BGF 320 compared to GFF (76).

Other studies were conducted to evaluate the effect of triple vs. double therapies on lung function.

TRILOGY was a randomized, parallel-group, double-blind, active-controlled study aimed to compare the efficacy of the single inhaler triple therapy vs. inhaled corticosteroid plus long-acting β2-agonist therapy [beclometasone dipropionate, formoterol fumarate, and glycopyrronium bromide (BDP/FF/GB) with beclometasone dipropionate and formoterol fumarate (BDP/FF)] over 52 weeks of treatment. Patients were 40 years or older, with a diagnosis of COPD, a FEV_1_ < 50%, a FEV_1_/FVC < 0·7, and at least one moderate or severe COPD exacerbation in the previous 12 months. Previous treatment included an inhaled corticosteroid plus a long-acting β2 agonist, or an inhaled corticosteroid plus a long-acting muscarinic antagonist, or a long-acting β2 agonist plus a long-acting muscarinic antagonist, or long-acting muscarinic antagonist monotherapy for at least 2 months before screening. Patients receiving triple therapy of an inhaled corticosteroid plus a long-acting β2 agonist plus a long-acting muscarinic antagonist were not eligible. Additionally, all patients needed to be symptomatic for inclusion, classified as a COPD Assessment Test (CAT) total score of 10 or more and a Baseline Dyspnea Index (BDI) focal score of 10 or less at screening, with the BDI criterion also confirmed at the randomization visit. 1,367 patients with symptomatic COPD have been enrolled. Three primary endpoints were assessed: change from baseline in pre-dose (morning) FEV_1_, change from baseline in 2-h post-dose FEV_1_, and Transition Dyspnea Index (TDI) focal score assessed at week 26. Results showed that BDP/FF/GB improved the morning pre-dose trough FEV_1_, and 2-h post dose FEV_1_ vs. ICS/LABA, and reduced exacerbations by 23% when compared with BDP/FF (77).

KRONOS study aimed to compare the efficacy of triple therapy with other dual therapies in patients with moderate to very severe COPD, with or without a history of exacerbations, over 24 weeks. This double-blind, parallel-group, multicenter phase 3 randomized controlled trial involved 1,902 patients. Participants were randomly assigned to receive triple therapy (budesonide/glycopyrronium /Formoterol Fumarate Metered Dose Inhaler -BGF MDI-) or dual therapies (glycopyrronium/formoterol fumarate [GFF] MDI and budesonide/formoterol fumarate [BFF] MDI, BUD/FORM DPI). BGF significantly improved morning pre-dose trough FEV_1_ when compared to GFF and BFF. This improvement was also observed for FEV_1_ AUC_0−4_. A significant reduction in the rate of moderate/severe exacerbations was observed in the triple therapy arm when compared to GFF (52% of reduction for moderate-to-severe exacerbation and 64% of reduction for severe exacerbations). Results showed that BGF MDI was more effective to improve FEV_1_ and to reduce exacerbation. Also, it was well-tolerated compared to the corresponding dual therapies among symptomatic patients with moderate to very severe COPD, with or without exacerbation history (78).

A *post-hoc* analysis (76) evaluated whether the benefits observed were driven by patients with ≥1 exacerbation in the 12 months prior to the study, considering that 74.4% were symptomatic patients without a prior history of exacerbations. Independently of eosinophil count, triple therapy results in exacerbations control in those symptomatic patients without exacerbations history. Respectively 52% and 58% of moderate/severe and severe exacerbations were reduced in this group, supporting the hypothesis that the exacerbations reduction in KRONOS was not driven by the subset of patients with a prior history of exacerbations (76).

## Discussion

Mortality represents the most important COPD outcome (17). In particular, cardiovascular diseases are important comorbidities in the prediction of all-cause mortality in COPD patients (31, 79).

So far, oxygen therapy, smoking discontinuation and surgical reduction of lung volume (in selected cases) were the only treatments able of reducing mortality.

Recently, the effectiveness of the triple inhaled therapy in the reduction of mortality was largely demonstrated, even though mortality was not the primary endpoint in the trials (80). The IMPACT study was the first large trial reporting significant mortality reduction data in patients who received triple inhaled therapy, with a hazard ratio for triple therapy vs. the dual bronchodilator umeclidinium/vilanterol of 0.58 (95% CI 0.38–0.88) and 0.61 (95% CI 0.40–0.93), although this finding derived from another endpoint (73, 74). Later on, the ETHOS study showed that the risk of death from any cause in the 320-μg budesonide triple therapy group was 49% lower than the glycopyrronium/formoterol group, including this analysis among key secondary endpoints (75, 76). For the reasons mentioned above, triple inhaled therapy was recommended in the latest GOLD guidelines as a potential option to reduce mortality in COPD patients with concomitant chronic diseases (57). Additionally, the choice of a device must be taken into account also evaluating the patient's clinical condition, as well as their ability to coordinate the inhalation maneuver and to generate sufficient inspiratory flow and their adherence to the treatment (81).

It is worth to note that previous large, controlled trials, comparing inhaled therapy with placebo, failed to demonstrate a significant effect on mortality. The TORCH trial aimed to evaluate the mortality reduction among patients with COPD treated with the combination of the long-acting b-agonist salmeterol and the inhaled corticosteroid fluticasone propionate compared with usual care (with each of the components alone) and with placebo over a 3-year period. 6,112 patients were enrolled in the study. The hazard ratio for death was 0.825 in the combination-therapy group (comparison to placebo) even if the reduction of death from all causes among patients with COPD in the combination-therapy group did not reach the predetermined level of statistical significance (82).

The SUMMIT trial was the largest randomized controlled COPD trial, where more than 16,000 patients with moderate airflow limitation (≥50% and ≤70% predicted forced expiratory volume in 1 s) and a history or an increased risk for cardiovascular disease were enrolled. The study aimed to compare the effectiveness of a combined treatment of fluticasone furoate and vilanterol compared with placebo. Results showed a non-significant reduction of all-cause mortality (12%) with ICS/LABA compared to placebo (83).

The UPLIFT trial evaluated the long-term effects of tiotropium therapy compared to placebo. The study included 5,993 COPD patients with a FEV1 of 70% or less after bronchodilation. Patients were evaluated in a 4-years follow up. Results showed a non-significant decrease in mortality (hazard ratio 0.89, 95% CI 0.79–1.02; *p* = 0.09) compared to placebo (84).

The OUTPUL study enrolled 18,615 patients and assessed whether in COPD patients adding an ICS to a long-acting bronchodilators (LB) would lead to a mortality reduction in patients discharged from hospital. Mortality rates were 110 and 143 cases per 1,000 person-years in the “LB plus ICS” and “LB alone” groups, respectively. The mortality reduction was much more pronounced in patients with frequent exacerbations, with a hazard ratio of 0.63 (95% CI: 0.44–0.90; *p* value: 0.012) (85).

Beneficial effects of triple inhaled therapy on the mortality reduction might be due to several mechanisms, such as reduction of hyperinflation, reduction of exacerbations risk, stabilization of atherosclerotic plaques and the improvement of cardiac perfusion.

COPD is characterized by a low-grade systemic inflammation (with the release of numerous proinflammatory cytokines and CRP) which can contribute to the pathogenesis of atherosclerosis and cardiovascular disease (86). In particular, high CRP levels are correlated with poor CV outcomes, such as atherosclerotic plaque genesis, rupture, and subsequent thrombofibrosis of vulnerable vessels. The efficacy of corticosteroids in the reduction of CRP and cytokines blood concentrations, which regulate CRP (such as interleukin 6), is well known. Especially, fluticasone demonstrated to be effective in the reduction of the serum CRP levels (66). ICSs are able to reduce the local inflammation and subsequent cardiovascular morbidity: Lofdahl et al. (87) demonstrated that budesonide was involved in the reduction of ischemic cardiac events, as well as angina pectoris, myocardial infarction and coronary artery disease. Moreover, ICS demonstrated to be involved in a reduction of the risk from myocardial infarction. This may be due to the reduction of COPD exacerbations, the reduction in systemic inflammation or the reduction in the adaptive immune response (88).

It is known that lung hyperinflation is associated with an increase in all-cause mortality in COPD patients. In particular, increased levels of static lung hyperinflation and emphysema are associated with a reduction in the cardiac chamber size and function, responsible of death for CV reasons. Triple inhaled therapies demonstrated to improve the right ventricular end-diastolic volume index and to reduce residual volume (89).

In conclusion, cardiovascular diseases represent an important comorbidity in the prediction of all-cause mortality in COPD patients. Numerous mechanisms may be involved in the development of COPD-related CVD: among these, a major role could be played by lung hyperinflation, hypoxemia, pulmonary hypertension (PH), systemic inflammation, oxidative stress, and exacerbations. The efficacy and safety of triple inhaled therapy were demonstrated by numerous controlled trials: above all, many robust data are now available on the effectiveness of the triple therapy to reduce mortality in COPD patients. Mechanisms involved need to be fully understood and they most likely include the reduction of hyperinflation, the improvement in the risk of exacerbations, the stabilization in the atherosclerotic plaques and the improvement of the cardiac perfusion. Thus, triple therapy may represent a valid option to reduce mortality in COPD patients, although it is still necessary to optimize the ideal patient profile.

## Author Contributions

PS and FP designed the project, selected the literature, and wrote and reviewed the manuscript. CA reviewed the manuscript. FR selected the literature and reviewed the manuscript. MB selected the literature and wrote the manuscript. LB designed the project and wrote the manuscript. All authors have reviewed and approved the manuscript.

## Funding

This study received funding from AstraZeneca. The funder was not involved in the study design, collection, analysis, interpretation of data, the writing of this article or the decision to submit it for publication.

## Conflict of Interest

The authors declare that the research was conducted in the absence of any commercial or financial relationships that could be construed as a potential conflict of interest.

## Publisher's Note

All claims expressed in this article are solely those of the authors and do not necessarily represent those of their affiliated organizations, or those of the publisher, the editors and the reviewers. Any product that may be evaluated in this article, or claim that may be made by its manufacturer, is not guaranteed or endorsed by the publisher.

## References

[1] Devine JF. Chronic obstructive pulmonary disease: an overview. Am Health Drug Benefits. (2008) 1:34–42. PMC4106574 25126252

[2] Singh D Agusti A Anzueto A Barnes PJ Bourbeau J Celli BR. Global strategy for the diagnosis, management, and prevention of chronic obstructive lung disease: the GOLD science committee report 2019. Eur Respir J. (2019) 53:1900164. 10.1183/13993003.00164-2019 30846476

[3] Barnes PJ. Chronic obstructive pulmonary disease. N Engl J Med. (2000) 343:269–80. 10.1056/NEJM200007273430407 10911010

[4] Pauwels RA Buist AS Calverley PM Jenkins CR Hurd SS GOLD Scientific Committee. Global strategy for the diagnosis, management, and prevention of chronic obstructive pulmonary disease NHLBI/WHO Global Initiative for Chronic Obstructive Lung Disease (GOLD) Workshop summary. Am J Respir Crit Care Med. (2001) 163:1256–76. 10.1164/ajrccm.163.5.2101039 11316667

[5] Organization WH. Chronic obstructive pulmonary disease (COPD) 2020. Available online at: https://www.who.int/news-room/fact-sheets/detail/chronic-obstructive-pulmonary-disease-(copd)

[6] Vijayan VK. Chronic obstructive pulmonary disease. Indian J Med Res. (2013) 137:251–69. 23563369 PMC3657849

[7] Rennard SI. Overview of causes of COPD. New understanding of pathogenesis and mechanisms can guide future therapy. Postgrad Med. (2002) 111:28–38. 10.3810/pgm.2002.06.1223 12082919

[8] Hogea SP Tudorache E Fildan AP Fira-Mladinescu O Marc M Oancea C. Risk factors of chronic obstructive pulmonary disease exacerbations. Clin Respir J. (2020) 14:183–97. 10.1111/crj.13129 31814260

[9] Silverman EK. Genetics of COPD. Annu Rev Physiol. (2020) 82:413–31. 10.1146/annurev-physiol-021317-121224 31730394 PMC7193187

[10] Albarrati AM Gale NS Munnery MM Cockcroft JR Shale DJ. Daily physical activity and related risk factors in COPD. BMC Pulm Med. (2020) 20:60. 10.1186/s12890-020-1097-y 32138714 PMC7059270

[11] Hikichi M Mizumura K Maruoka S Gon Y. Pathogenesis of chronic obstructive pulmonary disease (COPD) induced by cigarette smoke. J Thorac Dis. (2019) 11:S2129–40. 10.21037/jtd.2019.10.43 31737341 PMC6831915

[12] Wang Y Xu J Meng Y Adcock IM Yao X. Role of inflammatory cells in airway remodeling in COPD. Int J Chron Obstruct Pulmon Dis. (2018) 13:3341–8. 10.2147/COPD.S176122 30349237 PMC6190811

[13] Tkác J Man SF Sin DD. Systemic consequences of COPD. Ther Adv Respir Dis. (2007) 1:47–59. 10.1177/1753465807082374 19124347

[14] Ritchie AI Wedzicha JA. Definition, causes, pathogenesis, and consequences of chronic obstructive pulmonary disease exacerbations. Clin Chest Med. (2020) 41:421–38. 10.1016/j.ccm.2020.06.007 32800196 PMC7423341

[15] Global Initiative for Chronic Obstructive Lung Disease (GOLD). Global Strategy for the Diagnosis, Management, and Prevention of Chronic Obstructive Pulmonary Disease (2021 report).

[16] Negewo NA Gibson PG McDonald VM. COPD and its comorbidities: impact, measurement and mechanisms. Respirology. (2015) 20:1160–71. 10.1111/resp.12642 26374280

[17] Cazzola M MacNee W Martinez FJ Rabe KF Franciosi LG Barnes PJ. Outcomes for COPD pharmacological trials: from lung function to biomarkers. Eur Respir J. (2008) 31:416–69. 10.1183/09031936.00099306 18238951

[18] Sin DD Anthonisen NR Soriano JB Agusti AG. Mortality in COPD: role of co-morbidities. Eur Respir J. (2006) 28:1245–57. 10.1183/09031936.00133805 17138679

[19] Drummond MB Wise RA John M Zvarich MT McGarvey LP. Accuracy of death certificates in COPD: analysis from the TORCH trial. COPD. (2010) 7:179–85. 10.3109/15412555.2010.481695 20486816 PMC4802970

[20] World Health Organization. Chronic Obstructive Pulmonary Disease (COPD). (2019). Available online at: https://www.who.int/news-room/fact-sheets/detail/chronic-obstructive-pulmonary-disease-(copd) (accessed November 17, 2021).

[21] National National Institutes of Health and National Heart Lung and Blood Institute (NHLBI). Morbidity and Mortality: 2009 Chartbook on Cardiovascular, Lung, and Blood Diseases. NIH (2009).

[22] Berry CE Wise RA. Mortality in COPD: causes, risk factors, and prevention. COPD. (2010) 7:375–82. 10.3109/15412555.2010.510160 20854053 PMC7273182

[23] Bikov A Lange P Anderson JA Brook RD Calverley PMA Celli BR. FEV_1_ is a stronger mortality predictor than FVC in patients with moderate COPD and with an increased risk for cardiovascular disease. Int J Chron Obstruct Pulmon Dis. (2020) 15:1135–42. 10.2147/COPD.S242809 32547001 PMC7247606

[24] David S Edwards CW. Forced Expiratory Volume. Treasure Island, FL: StatPearls Publishing (2021). Available online at: https://www.ncbi.nlm.nih.gov/books/NBK540970/ (accessed September 14, 2020). 31082014

[25] Fletcher C Peto R. The natural history of chronic airflow obstruction. Br Med J. (1977) 1:1645–8. 871704 10.1136/bmj.1.6077.1645 PMC1607732

[26] Hoogendoorn M Hoogenveen RT Rutten-van Mölken MP Vestbo J Feenstra TL. Case fatality of COPD exacerbations: a meta-analysis and statistical modelling approach. Eur Respir J. (2011) 37:508–15. 10.1183/09031936.00043710 20595157

[27] Vieira EB Degani-Costa LH Amorim BC Oliveira LB Miranda-Silva T Sperandio PC. Modified BODE index to predict mortality in individuals with COPD: the role of 4-min step test. Respir Care. (2020) 65:977–83. 10.4187/respcare.06991 31992673

[28] Prudente R Frano E Mesquita C Ferrari R de Godoy I Tanni S. Predictors of mortality in patients with COPD after 9 years. Int J Chron Obstruct Pulmon Dis. (2018) 13:3389–98. 10.2147/COPD.S174665 30410324 PMC6198887

[29] Wood-Baker R Cochrane B Naughton MT. Cardiovascular mortality and morbidity in chronic obstructive pulmonary disease: the impact of bronchodilator treatment. Intern Med J. (2010) 40:94–101. 10.1111/j.1445-5994.2009.02109.x 19849745

[30] García Castillo E Alonso Pérez T Ancochea J Pastor Sanz MT Almagro P Martínez-Camblor P. Mortality prediction in chronic obstructive pulmonary disease comparing the GOLD 2015 and GOLD 2019 staging: a pooled analysis of individual patient data. ERJ Open Res. (2020) 6:00253–2020. 10.1183/23120541.00253-2020 33263033 PMC7682666

[31] Huiart L Ernst P Suissa S. Cardiovascular morbidity and mortality in COPD. Chest. (2005) 128:2640–6. 10.1378/chest.128.4.2640 16236937

[32] Magnussen H Watz H. Systemic inflammation in chronic obstructive pulmonary disease and asthma: relation with comorbidities. Proc Am Thorac Soc. (2009) 6:648–51. 10.1513/pats.200906-053DP 20008868

[33] Roversi S Fabbri LM Sin DD Hawkins NM Agustí A. Chronic obstructive pulmonary disease and cardiac diseases. An urgent need for integrated care. Am J Respir Crit Care Med. (2016) 194:1319–36. 10.1164/rccm.201604-0690SO 27589227

[34] Morgan AD Zakeri R Quint JK. Defining the relationship between COPD and CVD: what are the implications for clinical practice? Ther Adv Respir Dis. (2018) 12:1753465817750524. 10.1177/1753465817750524 29355081 PMC5937157

[35] Global Initiative for Chronic Obstructive Lung Disease (GOLD),. Global Strategy for the Diagnosis, Management, Prevention of Chronic Obstructive Pulmonary Disease (2018 Report). Available online at: https://goldcopd.org/wp-content/uploads/2017/11/GOLD-2018-v6.0-FINAL-revised-20-Nov_WMS.pdf (accessed November 28, 2017).

[36] Rossi A Aisanov Z Avdeev S Di Maria G Donner CF Izquierdo JL. Mechanisms, assessment and therapeutic implications of lung hyperinflation in COPD. Respir Med. (2015) 109:785–802. 10.1016/j.rmed.2015.03.010 25892293

[37] Parshall MB Schwartzstein RM Adams L Banzett RB Manning HL Bourbeau J. An official American Thoracic Society statement: update on the mechanisms, assessment, and management of dyspnea. Am J Respir Crit Care Med. (2012) 185:435–52. 10.1164/rccm.201111-2042ST 22336677 PMC5448624

[38] Rabe KF Hurst JR Suissa S. Cardiovascular disease and COPD: dangerous liaisons? [published correction appears in Eur Respir Rev. 2018 Nov 21;27(150)]. Eur Respir Rev. (2018) 27(149):180057. 10.1183/16000617.0057-2018 30282634 PMC9488649

[39] Kent BD Mitchell PD McNicholas WT. Hypoxemia in patients with COPD: cause, effects, and disease progression. Int J Chron Obstruct Pulmon Dis. (2011) 6:199–208. 10.2147/COPD.S10611 21660297 PMC3107696

[40] MacNee W. Pathophysiology of cor pulmonale in chronic obstructive pulmonary disease. Part One Am J Respir Crit Care Med. (1994) 150:833–52. 10.1164/ajrccm.150.3.8087359 8087359

[41] Vonk-Noordegraaf A. The shrinking heart in chronic obstructive pulmonary disease. N Engl J Med. (2010) 362:267–8. 10.1056/NEJMe0906251 20089979

[42] Zangiabadi A De Pasquale CG Sajkov D. Pulmonary hypertension and right heart dysfunction in chronic lung disease. Biomed Res Int. (2014) 2014:739674 10.1155/2014/739674 PMC4140123 25165714

[43] Rusinowicz T Zielonka TM Zycinska K. Cardiac arrhythmias in patients with exacerbation of COPD. Adv Exp Med Biol. (2017) 1022:53–62. 10.1007/5584_2017_41 28573445

[44] Bhatt SP Dransfield MT. Chronic obstructive pulmonary disease and cardiovascular disease. Transl Res. (2013) 162:237–51. 10.1016/j.trsl.2013.05.001 23727296

[45] Maclay JD MacNee W. Cardiovascular disease in COPD: mechanisms. Chest. (2013) 143:798–807. 10.1378/chest.12-0938 23460157

[46] André S Conde B Fragoso E Boléo-Tomé JP Areias V Cardoso J. GI DPOC-Grupo de Interesse na Doença Pulmonar Obstrutiva Crónica. COPD and Cardiovascular Disease. Pulmonology. (2019) 25:168–76. 10.1016/j.pulmoe.2018.09.006 30527374

[47] Song M Graubard BI Rabkin CS Engels EA. Neutrophil-to-lymphocyte ratio and mortality in the United States general population. Sci Rep. (2021) 11:464. 10.1038/s41598-020-79431-7 33431958 PMC7801737

[48] Almagro P Salvadó M Garcia-Vidal C Rodriguez-Carballeira M Delgado M Barreiro B. Recent improvement in long-term survival after a COPD hospitalisation. Thorax. (2010) 65:298–302. 10.1136/thx.2009.124818 20388752

[49] Suissa S Dell'Aniello S Ernst P. Long-term natural history of chronic obstructive pulmonary disease: severe exacerbations and mortality. Thorax. (2012) 67:957–63. 10.1136/thoraxjnl-2011-201518 22684094 PMC3505864

[50] Suissa S Hemmelgarn B Blais L Ernst P. Bronchodilators and acute cardiac death. Am J Respir Crit Care Med. (1996) 154:1598–602. 10.1164/ajrccm.154.6.8970341 8970341

[51] Maxwell SR Moots RJ Kendall MJ. Corticosteroids: do they damage the cardiovascular system? Postgrad Med J. (1994) 70:863–70. 7870631 10.1136/pgmj.70.830.863 PMC2398024

[52] Sholter DE Armstrong PW. Adverse effects of corticosteroids on the cardiovascular system. Can J Cardiol. (2000) 16:505–11. 10787466

[53] McKenzie DK Frith PA Burdon JG Town GI. The COPDX plan: Australian and New Zealand guidelines for the management of chronic obstructive pulmonary disease 2003. Med J Aust. (2003) 178(Suppl):S7–39. 10.5694/j.1326-5377.2003.tb05213.x 12633498

[54] SestiniPRenzoniERobinsonSPoolePRamFS. Short-acting beta 2 agonists for stable chronic obstructive pulmonary disease. Cochrane Database Syst Rev. (2002) 2002:CD001495. 10.1002/14651858.CD00149512519559

[55] Santus P Radovanovic D Di Marco S Valenti V Raccanelli R Blasi F. Effect of indacaterol on lung deflation improves cardiac performance in hyperinflated COPD patients: an interventional, randomized, double-blind clinical trial. Int J Chron Obstruct Pulmon Dis. (2015) 10:1917–23. 10.2147/COPD.S91684 26392766 PMC4574799

[56] Hohlfeld JM Vogel-Claussen J Biller H Berliner D Berschneider K Tillmann HC. Effect of lung deflation with indacaterol plus glycopyrronium on ventricular filling in patients with hyperinflation and COPD (CLAIM): a double-blind, randomised, crossover, placebo-controlled, single-centre trial. Lancet Respir Med. (2018) 6:368–78. 10.1016/S2213-2600(18)30054-7 29477448

[57] Schols AM Wesseling G Kester AD de Vries G Mostert R Slangen J. Dose dependent increased mortality risk in COPD patients treated with oral glucocorticoids. Eur Respir J. (2001) 17:337–42. 10.1183/09031936.01.17303370 11405508

[58] Petite SE. What is the role of triple inhaled therapy in COPD? JAAPA. (2019) 32:44–5. 10.1097/01.JAA.0000580568.79593.55 31567741

[59] Global Initiative for Chronic Obstructive Lung Disease (GOLD),. Global Strategy for the Diagnosis, Management, Prevention of Chronic Obstructive Pulmonary Disease (2021 report). Available online at: https://goldcopd.org/wp-content/uploads/2020/11/GOLD-REPORT-2021-v1.1-25Nov20_WMV.pdf

[60] Zheng Y Zhu J Liu Y Lai W Lin C Qiu K. Triple therapy in the management of chronic obstructive pulmonary disease: systematic review and meta-analysis. BMJ. (2018) 363:k4388. 10.1136/bmj.k4388 30401700 PMC6218838

[61] Trixeo. Summary of Product Characteristics. Available online at: https://www.ema.europa.eu/en/medicines/human/EPAR/trixeo-aerosphere

[62] Trimbow. Summary of Product Characteristics. Available online at: https://www.ema.europa.eu/en/medicines/human/EPAR/trimbow

[63] Trelegy. Summary of Product Characteristics. Available online at: https://www.ema.europa.eu/en/medicines/human/EPAR/trelegy-ellipta

[64] Vanfleteren L Fabbri LM Papi A Petruzzelli S Celli B. Triple therapy (ICS/LABA/LAMA) in COPD: time for a reappraisal. Int J Chron Obstruct Pulmon Dis. (2018) 13:3971–81. 10.2147/COPD.S185975 30587953 PMC6296179

[65] Aksoy MO Mardini IA Yang Y. Glucocorticoid effects on the beta-adrenergic receptor-adenylyl cyclase system of human airway epithelium. J Allergy Clin Immunol. (2002) 109:491–7. 10.1067/mai.2002.122154 11897997

[66] Sin DD Lacy P York E Man SF. Effects of fluticasone on systemic markers of inflammation in chronic obstructive pulmonary disease. Am J Respir Crit Care Med. (2004) 170:760–5. 10.1164/rccm.200404-543OC 15229100

[67] Barnes PJ. Muscarinic receptor subtypes in airways. Life Sci. (1993) 52:521–7. 8441331 10.1016/0024-3205(93)90310-y

[68] Barnes PJ. Chronic Obstructive Pulmonary Disease. London: Chapman and Hall (1995).

[69] Montuschi P Malerba M Macis G Mores N Santini G. Triple inhaled therapy for chronic obstructive pulmonary disease. Drug Discov Today. (2016) 21:1820–7. 10.1016/j.drudis.2016.07.009 27452453

[70] Bourbeau J Bafadhel M Barnes NC Compton C Di Boscio V Lipson DA. Benefit/risk profile of single-inhaler triple therapy in COPD. Int J Chron Obstruct Pulmon Dis. (2021) 16:499–517. 10.2147/COPD.S291967 33688176 PMC7935340

[71] Papi A Vestbo J Fabbri L Corradi M Prunier H Cohuet G. Extrafine inhaled triple therapy versus dual bronchodilator therapy in chronic obstructive pulmonary disease (TRIBUTE): a double-blind, parallel group, randomised controlled trial [published correction appears in Lancet. 2018 Feb 26]. Lancet. (2018) 391:1076–84. 10.1016/S0140-6736(18)30206-X 29429593

[72] Vestbo J Fabbri L Papi A Petruzzelli S Scuri M Guasconi A. Inhaled corticosteroid containing combinations and mortality in COPD. Eur Respir J. (2018) 52:1801230. 10.1183/13993003.01230-2018 30209195

[73] Lipson DA Barnhart F Brealey N Brooks J Criner GJ Day NC. Once-daily single-inhaler triple versus dual therapy in patients with COPD. N Engl J Med. (2018) 378:1671–80. 10.1056/NEJMoa1713901 29668352

[74] Lipson DA Crim C Criner GJ Day NC Dransfield MT Halpin DMG. Reduction in all-cause mortality with fluticasone furoate/umeclidinium/vilanterol in patients with chronic obstructive pulmonary disease. Am J Respir Crit Care Med. (2020) 201:1508–16. 10.1164/rccm.201911-2207OC 32162970 PMC7301738

[75] Rabe KF Martinez FJ Ferguson GT Wang C Singh D Wedzicha JA. Triple inhaled therapy at two glucocorticoid doses in moderate-to-very-severe COPD. N Engl J Med. (2020) 383:35–48. 10.1056/NEJMoa1916046 32579807

[76] Martinez FJ Ferguson GT Bourne E Ballal S Darken P Aurivillius M. Budesonide/glycopyrrolate/formoterol fumarate metered dose inhaler improves exacerbation outcomes in patients with COPD without a recent exacerbation history: a subgroup analysis of KRONOS. Int J Chron Obstruct Pulmon Dis. (2021) 16:179–89. 10.2147/COPD.S286087 33542624 PMC7851632

[77] Singh D Papi A Corradi M Pavlišová I Montagna I Francisco C. Single inhaler triple therapy versus inhaled corticosteroid plus long-acting β2-agonist therapy for chronic obstructive pulmonary disease (TRILOGY): a double-blind, parallel group, randomised controlled trial. Lancet. (2016) 388:963–73. 10.1016/S0140-6736(16)31354-X 27598678

[78] Ferguson GT Rabe KF Martinez FJ Fabbri LM Wang C Ichinose M. Triple therapy with budesonide/glycopyrronium/formoterol fumarate with co-suspension delivery technology versus dual therapies in chronic obstructive pulmonary disease (KRONOS): a double-blind, parallel-group, multicentre, phase 3 randomised controlled trial [published correction appears in *Lancet Respir Med*. 2018 Oct 4] [published correction appears in *Lancet Respir Med*. 2019;7(2):e9]. Lancet Respir Med. (2018) 6:747–58. 10.1016/S2213-2600(18)30327-8 30232048

[79] Divo M Cote C de Torres JP Casanova C Marin JM Pinto-Plata V. Comorbidities and risk of mortality in patients with chronic obstructive pulmonary disease. Am J Respir Crit Care Med. (2012) 186:155–61. 10.1164/rccm.201201-0034OC 22561964

[80] Andreas S Taube C. Inhaled therapy reduces COPD mortality. ERJ Open Res. (2020) 6:00634–2020. 10.1183/23120541.00634-2020 33294425 PMC7701338

[81] Scichilone N Benfante A Bocchino M Braido F Paggiaro P Papi A. Which factors affect the choice of the inhaler in chronic obstructive respiratory diseases? Pulm Pharmacol Ther. (2015) 31:63–7. 10.1016/j.pupt.2015.02.006 25724817

[82] Calverley PM Anderson JA Celli B Ferguson GT Jenkins C Jones PW. Salmeterol and fluticasone propionate and survival in chronic obstructive pulmonary disease. N Engl J Med. (2007) 356:775–89. 10.1056/nejmoa063070 17314337

[83] Vestbo J Anderson JA Brook RD Calverley PM Celli BR Crim C. Fluticasone furoate and vilanterol and survival in chronic obstructive pulmonary disease with heightened cardiovascular risk (SUMMIT): a double-blind randomised controlled trial. Lancet. (2016) 387:1817–26. 10.1016/S0140-6736(16)30069-1 27203508

[84] Tashkin DP Celli B Senn S Burkhart D Kesten S Menjoge S. A 4-year trial of tiotropium in chronic obstructive pulmonary disease. N Engl J Med. (2008) 359:1543–54. 10.1056/nejmoa0805800 18836213

[85] Di Martino M Agabiti N Cascini S Kirchmayer U Bauleo L Fusco D. The effect on total mortality of adding inhaled corticosteroids to long-acting bronchodilators for COPD: a real practice analysis in Italy. COPD. (2016) 13:293–302. 10.3109/15412555.2015.1044861 26514912

[86] Sin DD Man SF. Why are patients with chronic obstructive pulmonary disease at increased risk of cardiovascular diseases? The potential role of systemic inflammation in chronic obstructive pulmonary disease. Circulation. (2003) 107:1514–9. 10.1161/01.CIR.0000056767.69054.B3 12654609

[87] Löfdahl CG Postma DS Pride NB Boe J Thorén A. Possible protection by inhaled budesonide against ischaemic cardiac events in mild COPD. Eur Respir J. (2007) 29:1115–9. 10.1183/09031936.00128806 17331963

[88] Macie C Wooldrage K Manfreda J Anthonisen NR. Inhaled corticosteroids and mortality in COPD. Chest. (2006) 130:640–6. 10.1378/chest.130.3.640 16963657

[89] Stone IS Barnes NC James WY Midwinter D Boubertakh R Follows R. Lung deflation and cardiovascular structure and function in chronic obstructive pulmonary disease. A Randomized Controlled Trial. Am J Respir Crit Care Med. (2016) 193:717–26. 10.1164/rccm.201508-1647OC 26550687 PMC5440091

